# Learning Potential as a Dynamic Component of Academic Intelligence: An fNIRS-Based Dynamic Assessment Study of English Reading Comprehension

**DOI:** 10.3390/jintelligence14080180

**Published:** 2026-08-06

**Authors:** Jingying Chen, Yijia Zheng, Yuetong Li, Xiaodi Liu, Rui Li

**Affiliations:** 1 Educational Development Research Center of Southern Xinjiang, School of Education Science, Kashi University, Kashgar 844000, China; 2 National Engineering Research Center of Educational Big Data, Central China Normal University, Wuhan 430079, China

**Keywords:** dynamic assessment, learning potential, English reading comprehension, functional near-infrared spectroscopy (fNIRS), cognitive activation

## Abstract

Foreign language reading comprehension is a critical indicator of university students’ academic cognitive ability and global competence, yet effectively assessing their latent learning potential remains a major challenge. Therefore, from a neurocognitive perspective, this study combined functional near-infrared spectroscopy (fNIRS) with dynamic assessment (DA) to investigate the activation characteristics of the left frontal and temporal regions in non-English majors during reading tasks across two difficulty levels (CET-4/CET-6) and two assessment conditions (static/dynamic testing). The results showed that reading tasks activated the dorsolateral prefrontal cortex and middle temporal gyrus. Compared with CET-4, the more difficult CET-6 task showed a broader descriptive cortical activation pattern. Notably, mediated prompts were associated with different behavioral and activation-frequency patterns across task difficulty levels: in the CET-4 task, dynamic assessment showed higher normalized activation-frequency values than static assessment in areas corresponding to the middle and inferior frontal gyrus, accompanied by rising prompt scores; however, the CET-6 task exhibited the opposite pattern. These findings suggest that mediated prompts may be more effective in stimulating learning potential when task difficulty aligns with the learner’s zone of proximal development. Overall, by considering behavioral dynamic-assessment indicators in foreign-language reading together with fNIRS-based cortical activation patterns, this exploratory study offers preliminary insights into learning potential, personalized instructional interventions, and academic cognitive assessment.

## 1. Introduction

Language ability is a core medium of social interaction and a major manifestation of cognitive competence in academic learning and cross-cultural communication. For Chinese college students, English reading comprehension requires semantic processing, working memory, attentional control, inference, and metacognitive monitoring (Aziz & Rawian, 2022). Conventional foreign-language assessment is typically static and summative (Teo, 2014). Although such an assessment is useful for standardizing and comparing current performance, it provides limited information about what learners may achieve when appropriate assistance is provided (Zhao & Aryadoust, 2025).

Dynamic assessment (DA) offers a theoretically grounded way to address this limitation. Based on Vygotsky’s concept of the zone of proximal development (ZPD), DA provides mediated prompts during assessment and examines how learners respond to graduated assistance (Vygotsky, 1978). In this sense, DA is not only a method for measuring existing ability but also a way to estimate learning potential (Brown & French, 1979). This developmental orientation is closely related to the study of intelligence, particularly to research on modifiability, teachability, and context-sensitive measurement (Tzuriel, 2000). In the present study, these related terms are used at different conceptual levels. Academic intelligence refers to the broader domain of cognitive performance in educational contexts. Cognitive modifiability and plasticity provide the theoretical basis for expecting performance to change under appropriate support. Learning potential refers more specifically to the learner’s observable responsiveness to mediation within a task. Dynamic assessment is the procedure used to elicit and measure this responsiveness through graduated prompts. Recent work has extended dynamic assessment beyond traditional teacher mediation (Poehner & Wang, 2021). Poehner et al. applied computerized dynamic assessment to diagnose L2 reading and listening development (Poehner et al., 2015); Behbahani and Karimpour examined mobile-mediated dynamic assessment of L2 reading (Behbahani & Karimpour, 2025); Piyumi Udeshinee et al. redesigned a DA regulatory scale for synchronous text-chat environments involving teachers (Piyumi Udeshinee et al., 2024); and Zhang et al. used human–computer interactive games for the dynamic assessment of language skills (Zhang et al., 2024). Together, these studies indicate that DA now spans computerized, mobile-mediated, collaborative, and game-based learning contexts. In L2 reading research, Yang and Qian examined dynamic assessment of EFL reading proficiency (Yang & Qian, 2017, 2023), Zadkhast et al. compared concurrent and cumulative group dynamic assessment for reading micro-skills (Zadkhast et al., 2023), and Jin and Liu used hybrid computerized dynamic assessment to diagnose and promote L2 inferential reading development (Jin & Liu, 2026). These studies show that interventionist, cumulative, and hybrid computerized DA approaches have been used to assess reading skills, inferential comprehension, and learners’ developmental potential (Siengyen & Wasanasomsithi, 2024). Brown and French argued that learning potential can be identified through the ZPD, and Tzuriel emphasized that DA integrates assessment with educational intervention.

Existing DA studies in second-language learning have primarily inferred learning potential from behavioral scores, prompt levels, and response accuracy (Jia et al., 2023; Kozulin & Garb, 2003). These indicators are important, but they do not reveal the internal neurocognitive processes through which learning potential is activated (Sun et al., 2023). The same behavioral gain may reflect improved semantic integration, reduced working-memory burden, enhanced attentional control, or simple familiarity with the task (Rovetti et al., 2021). Evidence from Binder and Desai’s review of semantic memory, Marks et al.’s study of morphological awareness in Spanish–English bilingual children (Marks et al., 2022), Babayiğit et al.’s work on vocabulary limitations in bilingual reading comprehension, and Aziz and Rawian’s model of higher-order thinking and metacognitive awareness indicates that reading comprehension draws on semantic memory, vocabulary knowledge, morphological awareness, and higher-order reasoning (Binder & Desai, 2011; Babayiğit et al., 2022). A single score is therefore unlikely to explain the sources of performance differences adequately. Therefore, combining DA with neurocognitive measurement can extend learning-potential research from a purely behavioral approach to an integrated framework linking performance, brain activation, and instructional intervention.

Functional near-infrared spectroscopy (fNIRS) provides a feasible tool for this purpose. fNIRS is a non-invasive neuroimaging technique that uses near-infrared light to detect changes in oxygenated hemoglobin (HbO) and deoxygenated hemoglobin (HbR), thereby estimating cortical activation (Jöbsis, 1977). Da Silva Soares et al. combined fNIRS with eye tracking in a naturalistic educational setting to examine children’s cognitive effort during mental rotation (Da Silva Soares et al., 2022), and Li et al. used fNIRS hyperscanning to investigate teacher–student interaction in language learning under anxiety-inducing conditions (Li et al., 2025). These studies show that fNIRS can measure cortical oxygenation in relatively natural learning and language contexts and can therefore complement behavioral measures. Language-related fNIRS studies provide converging evidence for the involvement of frontal and temporal language networks. Butler et al. examined the production and comprehension of semantically and phonologically related words (Butler et al., 2023), Luo et al. localized functional specialization in inferior frontal language regions (Luo et al., 2025), Jiang et al. investigated private speech during L2 oral production (Jiang et al., 2025), and He and Hu compared functional connectivity during Chinese–English simultaneous translation (He & Hu, 2022). These findings link prefrontal, inferior frontal, temporal, and other language-related regions to lexical–semantic processing, language production, communicative regulation, and bilingual switching. In cognitive-load and educational-task research, Rovetti et al. used fNIRS to compare verbal working-memory load across auditory and visual modalities (Rovetti et al., 2021), Hamann and Carstengerdes combined fNIRS with EEG to examine workload-induced cortical oxygenation during simulated flight (Hamann & Carstengerdes, 2022), Čeko et al. captured cortical processing during reading (Čeko et al., 2024), and Akincioglu et al. classified English reading comprehension status from fNIRS signals (Akincioglu et al., 2025). These studies indicate that fNIRS can index changes in working-memory load, mental workload, and reading comprehension states, providing physiological evidence for task-difficulty effects. Compared with fMRI, fNIRS allows for more naturalistic task settings and is less sensitive to environmental noise; compared with EEG, it offers better spatial localization. These features make fNIRS suitable for educational and language-learning research. Previous studies have shown that frontal and temporal regions are involved in language comprehension, semantic integration, working memory, and cognitive load.

The present study used College English Test Band 4 (CET-4) and Band 6 (CET-6) reading materials to represent two levels of task difficulty, introduced mediated prompts within a DA framework, and recorded cortical activation in left frontal and temporal regions using fNIRS. The study addressed three questions: (1) Do English reading tasks of different difficulties elicit different cortical activation patterns? (2) Do mediated prompts modulate brain activation and behavioral performance during reading comprehension? (3) Can fNIRS provide neurocognitive evidence for the dynamic assessment of learning potential beyond behavioral scores? By answering these questions, the study situates foreign-language reading assessment within a broader framework of learning potential, cognitive plasticity, and neuroimaging-based evidence.

## 2. Materials and Methods

### 2.1. Participants

Thirty non-English-major college students were recruited from Central China Normal University. Participants were included if they were non-English majors, aged 18–30, right-handed, and had normal or corrected-to-normal vision sufficient for reading computer-presented materials. Their English reading vocabulary size was above 4000 words, as measured by the Baicizhan vocabulary test within the Baicizhan App (version 7.6.3; Chengdu Chaoai Learning Technology Co., Ltd., Chengdu, China). This criterion was used to ensure that participants possessed sufficient basic lexical knowledge for completing the English reading comprehension tasks, rather than to provide a comprehensive measure of English proficiency. All participants reported no history of neurological or language disorders. The experiment was conducted after obtaining the informed consent of the participants and the study was approved by the institutional ethics committee. Because fNIRS recording is sensitive to head movement, optode contact, and ambient light, seven participants were excluded after data preprocessing. The final valid sample included 23 participants, including 5 males and 18 females. Invalid data were mainly caused by large head or body movements, poor channel quality, individual differences in scalp–optode contact, or interference from laboratory lighting.

### 2.2. Experimental Design

A within-subject design was adopted, with reading difficulty (CET-4 vs. CET-6 reading) and assessment condition (static assessment vs. dynamic assessment) as the two factors. Reading difficulty was used to manipulate cognitive load, and assessment condition was used to compare independent performance without assistance with learning potential activated by mediated prompts. All participants completed reading tasks at both difficulty levels, and the order of CET-4 and CET-6 tasks was randomized. Dynamic assessment was administered only for items answered incorrectly in the preceding static assessment. Therefore, for participants who answered all items correctly in a static task, the corresponding dynamic-test data were incomplete, and fNIRS analyses of dynamic phases were based on available valid task data. Thus, the static and dynamic phases were based on different sets of observations, and their activation-frequency maps were compared descriptively.

### 2.3. fNIRS Recording System and Optode Placement

**Figure 1:**
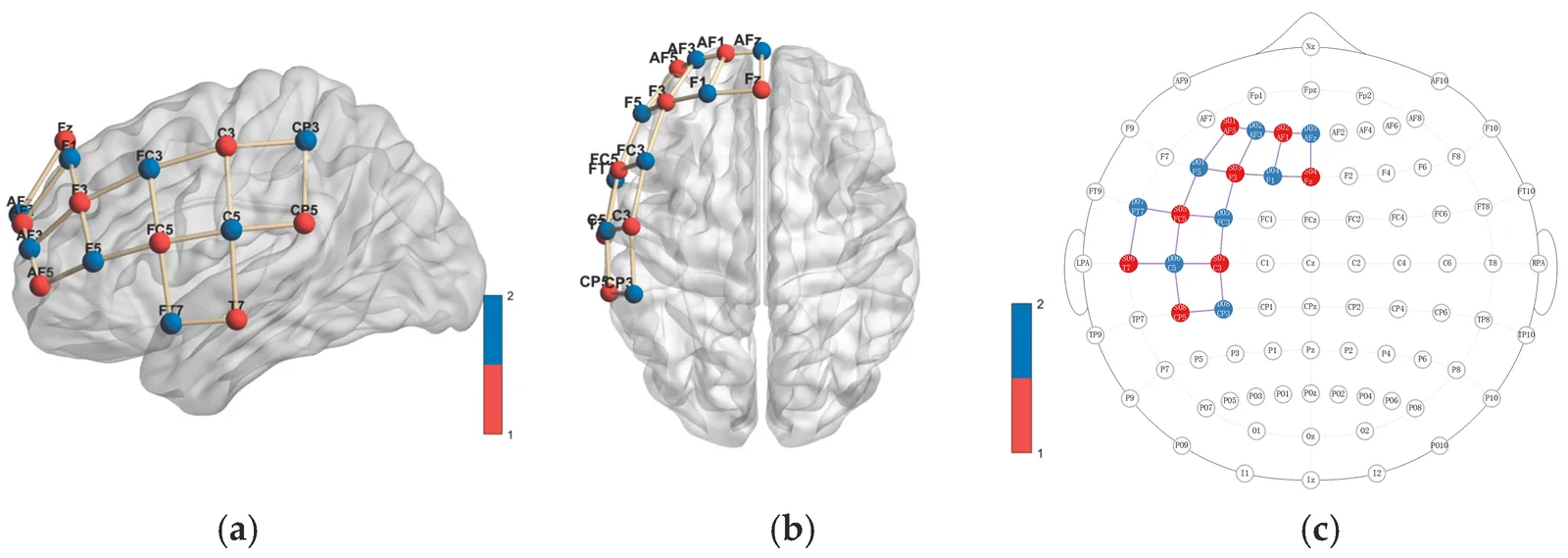
(**a**) Left view: fNIRS optode placement; (**b**) top view: fNIRS optode placement; (**c**) fNIRS channel layout.

Cortical hemodynamic activity was recorded using a NIRScoutX 64 × 32 fNIRS system (NIRx Medizintechnik GmbH, Berlin, Germany) with a sampling rate of 7.812 Hz. Data were acquired using NIRStar version 15.3 (NIRx Medizintechnik GmbH, Berlin, Germany). Two wavelengths of near-infrared light, 785 nm and 830 nm, were used to measure relative changes in HbO and HbR. The probe array consisted of 8 emitters and 8 detectors, forming 22 channels over the left frontal and temporal cortices. The montage mainly covered regions relevant to English reading comprehension, including the dorsolateral prefrontal cortex (DLPFC), middle frontal gyrus (MFG), inferior frontal gyrus (IFG), precentral gyrus (PreCG), middle temporal gyrus (MTG), and supramarginal gyrus (Čeko et al., 2024). The optode placement and channel layout are presented in Figure 1, and the MNI coordinates are provided in Appendix A. The montage visualization and MNI coordinates were generated using NIRSite version 2021.4.0.2 (NIRx Medizintechnik GmbH, Berlin, Germany).

### 2.4. Reading Materials and Rationale for Using CET-4 and CET-6

The reading materials were selected from College English Test Band 4 (CET-4) and Band 6 (CET-6), which represent moderate and relatively high levels of English reading difficulty for Chinese college students. The College English Test is a large-scale standardized national English examination widely used in Chinese higher education, especially for non-English majors. CET-4 corresponds to the general requirements of college English learning, whereas CET-6 corresponds to higher-level requirements. Reading comprehension is a core component of the CET score. Therefore, using CET reading materials provides a standardized, pedagogically meaningful, and ecologically valid way to operationalize reading difficulty in Chinese college students.

Using CET-4 and CET-6 as difficulty levels is also theoretically meaningful for DA. The goal of DA is not merely to compare high and low scores but to determine whether a task falls within a learner’s ZPD. If learners improve after mediated prompts under an appropriately challenging condition, the task may lie within their ZPD (Ajideh & Nourdad, 2012). If the task is excessively difficult, learning potential may not be effectively activated even with prompts. Thus, CET-4 and CET-6 materials served not only as language stimuli but also as a difficulty gradient for testing the effectiveness and boundary conditions of mediation. A pilot difficulty rating confirmed that CET-4 materials were perceived as significantly less difficult than CET-6 materials (M_CET-4 = 5.31, M_CET-6 = 6.42, *p* < 0.001).

### 2.5. Mediated Prompts and Scoring of Learning Potential

An English reading comprehension dynamic assessment system was custom-developed by the research team using Python 3.10 (Python Software Foundation, Beaverton, OR, USA) according to ZPD theory and the reading requirements of the CET (Poehner & Lantolf, 2013). Mediated prompts were designed from implicit to explicit in three levels: first, informing the learner that the answer was incorrect; second, providing information related to the correct answer; and third, providing more specific cues related to the correct answer. More implicit prompts indicate that the learner is closer to independent problem solving, whereas more explicit prompts indicate greater need for external support. The prompt levels and scoring rules are shown in Table 1.

Behavioral indicators included actual score, prompt score, mediated score, and learning potential score (LPS). The actual score refers to performance without prompts; the prompt score refers to scores obtained after mediated prompts during incorrect-item reattempts; and the mediated score is the sum of the actual score and prompt score. In this study, learning potential was operationalized behaviorally as learners’ responsiveness to graduated mediation, rather than as a latent construct directly measured by fNIRS. LPS was used as a normalized index of mediated performance: LPS = mediated score/maximum possible score. Thus, LPS and prompt scores were interpreted as behavioral indicators of responsiveness to mediation, whereas fNIRS activation was interpreted as supplementary process-level information associated with task difficulty and mediated reattempts.

### 2.6. Procedure

**Figure 2:**
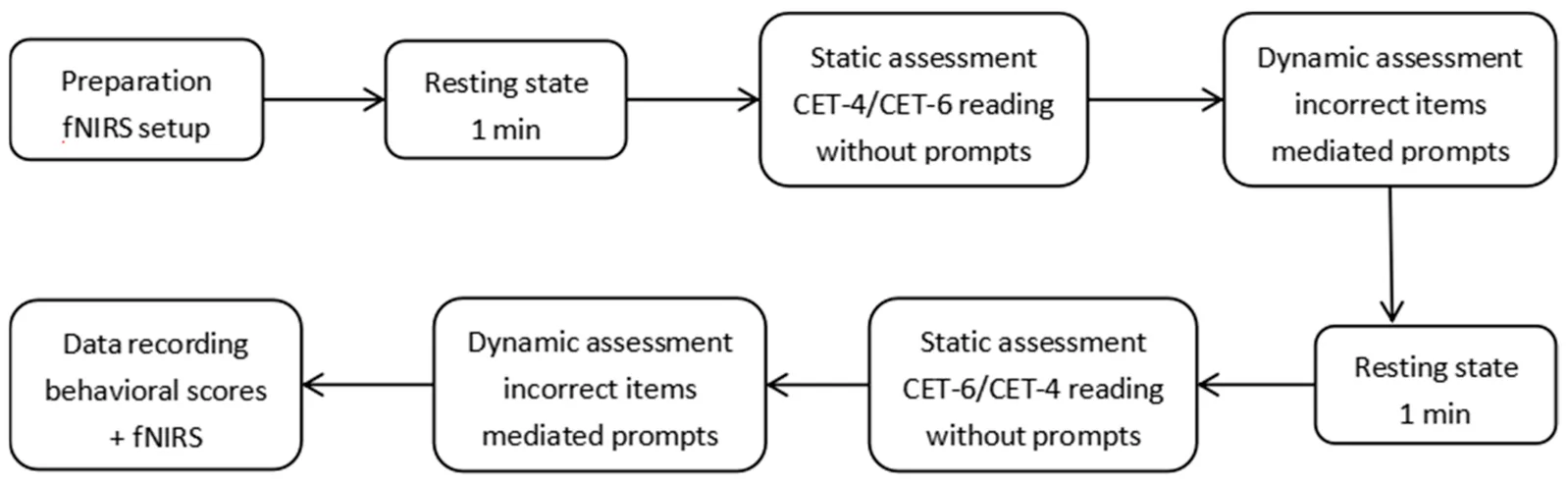
Experimental procedure.

The experiment consisted of CET-4 and CET-6 reading tasks, each including a static assessment phase and a dynamic assessment phase. Before each reading task, participants completed a 1-min eyes-closed resting-state period. They then entered the static assessment phase and independently answered five multiple-choice reading comprehension questions without prompts. Immediately after the static phase, participants entered the dynamic assessment phase. Only items answered incorrectly in the static phase entered this phase. During each mediated reattempt, the original reading passage and the prompt information were displayed on the left side of the screen, and the incorrectly answered question was displayed on the right side. Participants could review the passage while considering the prompt. No fixed time limit was imposed for each prompt; participants proceeded at their own pace in order to approximate natural learning conditions. Prompts were standardized across participants, and the same prompt content was presented for the same item and prompt level. The prompt hierarchy was developed according to professional guidance materials for English reading instruction and was checked in a pilot procedure. The order of CET-4 and CET-6 tasks was randomized to reduce order effects. The experimental procedure is shown in Figure 2.

Across the valid sample, 93 incorrectly answered items entered the dynamic assessment phase. For CET-4 items, 30 incorrect items entered dynamic assessment: 12 required no prompt, 15 received a Level 1 prompt, and 3 received a Level 2 prompt. For CET-6 items, 63 incorrect items entered dynamic assessment: 22 required no prompt, 33 received a Level 1 prompt, and 8 received a Level 2 prompt.

### 2.7. fNIRS Preprocessing and Statistical Analysis

fNIRS data were preprocessed using nirsLAB_v201904_64bit (NIRx Medizintechnik GmbH, Berlin, Germany), MATLAB R2024a (The MathWorks, Inc., Natick, MA, USA), and Microsoft Excel 2021 (Microsoft Corporation, Redmond, WA, USA) to obtain HbO, HbR, and total hemoglobin data for each channel and participant. The 1-min eyes-closed resting period before each reading task served as the baseline for both the static and dynamic phases, and task periods were defined by the recorded start and end markers. Discontinuities and spike artifacts were addressed with the Remove Discontinuities and Remove Spike Artifacts functions in nirsLAB, followed by filtering before data export. Channel quality was assessed with the Good/Bad Channel module, with Gain Setting set to 8 and the CV threshold set to 7.5%. According to the neurovascular coupling principle underlying fNIRS, task execution is typically associated with increased HbO and decreased HbR (Jöbsis, 1977). In the present exploratory analysis, activation index, activation magnitude, and activation frequency refer to different quantities. For each analyzable participant–condition record, the channel-level activation index was calculated as the absolute difference in the HbO-HbR contrast between the task phase and the corresponding resting phase. The HbO-HbR contrast was used because task-related HbO and HbR typically change in opposite directions, and the absolute value provided a direction-independent basis for ranking channels. Activation magnitude refers only to the numerical size of this channel-level index and is not used to describe the regional frequency maps. Because the present study aimed to provide an exploratory descriptive fNIRS summary rather than confirmatory neuroimaging inference, no full GLM-based channel-wise inference was performed. In addition, because the dynamic-assessment phase was contingent on errors in the preceding static phase, the number of valid dynamic observations varied across participants and items. Therefore, activation indices and top-channel frequencies were used as descriptive summaries of relative hemodynamic patterns rather than as confirmatory channel-wise or ROI-level evidence. Future studies should apply GLM analysis, motion-artifact correction procedures, functional connectivity analysis, and correction for multiple comparisons to further validate the present findings.

For behavioral data, paired-sample *t*-tests were performed using Python 3.10 to compare actual scores from static assessment with mediated scores after dynamic assessment separately for the CET-4 and CET-6 conditions. For each comparison, within-participant difference scores were defined as actual score minus mediated score, and Cohen’s dz was calculated by dividing the absolute mean of the difference scores by their standard deviation. Pearson correlations were also calculated using Python 3.10 among actual score, prompt score, mediated score, and LPS. For fNIRS data, mean HbO and activation indices were first calculated for each channel. Channels were then ranked by activation level for each participant, and the occurrence frequencies of the top 50% activated channels were obtained by assigning one occurrence to each of the source and detector optode locations, forming a retained channel and summing the occurrences at each optode location across all available participant records within a condition. The resulting optode-level frequencies were mapped to the corresponding cortical regions. Within-condition maximum normalization was then applied separately to each condition. The largest optode-level frequency within each condition served as the common denominator for all optode locations in that condition. Thus, all CET-4 static frequencies were divided by 37 (the maximum at C5), all CET-4 dynamic frequencies by 29 (the shared maximum at AF3 and C5), all CET-6 static frequencies by 35 (the maximum at T7), and all CET-6 dynamic frequencies by 38 (the maximum at F1). This rescaled the maximum in each condition to 1 and the remaining values to between 0 and 1. After normalization, the static and dynamic normalized frequencies at the same optode location were paired, and one difference value was calculated for that location. For CET-4 (dynamic minus static), a positive value indicates a higher normalized activation frequency in the dynamic condition, whereas a negative value indicates a higher value in the static condition. For CET-6 (static minus dynamic), a positive value indicates a higher normalized activation frequency in the static condition, whereas a negative value indicates a higher value in the dynamic condition. In both comparisons, zero indicates equal normalized activation frequencies in the two conditions.

## 3. Results

### 3.1. Behavioral Results

**Figure 3:**
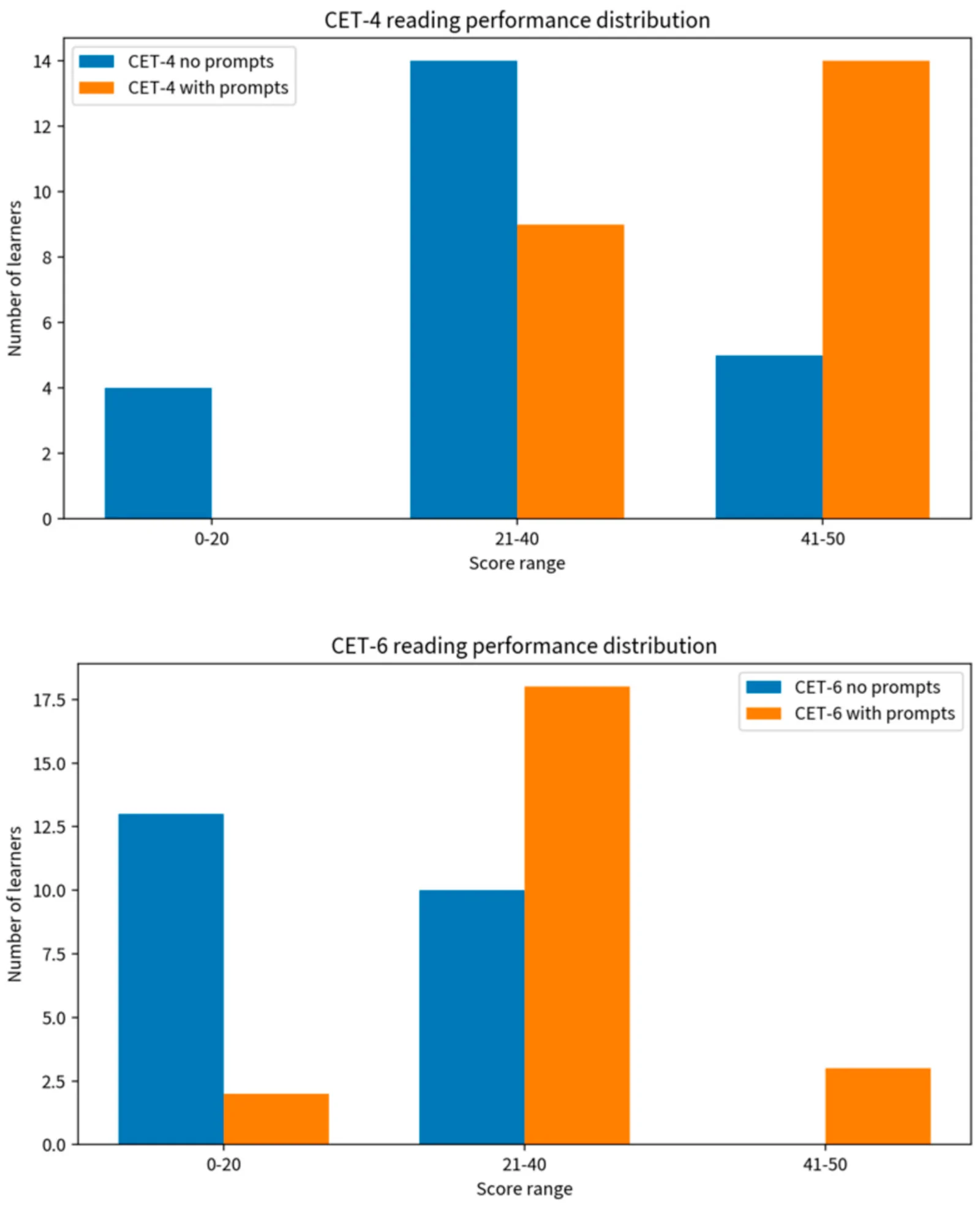
Performance distributions before and after prompts in CET-4 and CET-6 reading.

Paired-sample *t*-tests showed that mediated scores after dynamic assessment were significantly higher than actual scores from static assessment in both reading conditions (CET-4: t(22) = −5.057, *p* < 0.001, Cohen’s dz = 1.05; CET-6: t(22) = −7.036, *p* < 0.001, Cohen’s dz = 1.47). Without prompts, the mean score was 45 for CET-4 reading and 30 for CET-6 reading. With prompts, the mean score increased to 47 for CET-4 and 41 for CET-6. Score distributions further showed that learners shifted from lower score ranges to higher score ranges after prompts in both tasks, indicating that mediation generally improved reading performance (Figure 3).

**Figure 4:**
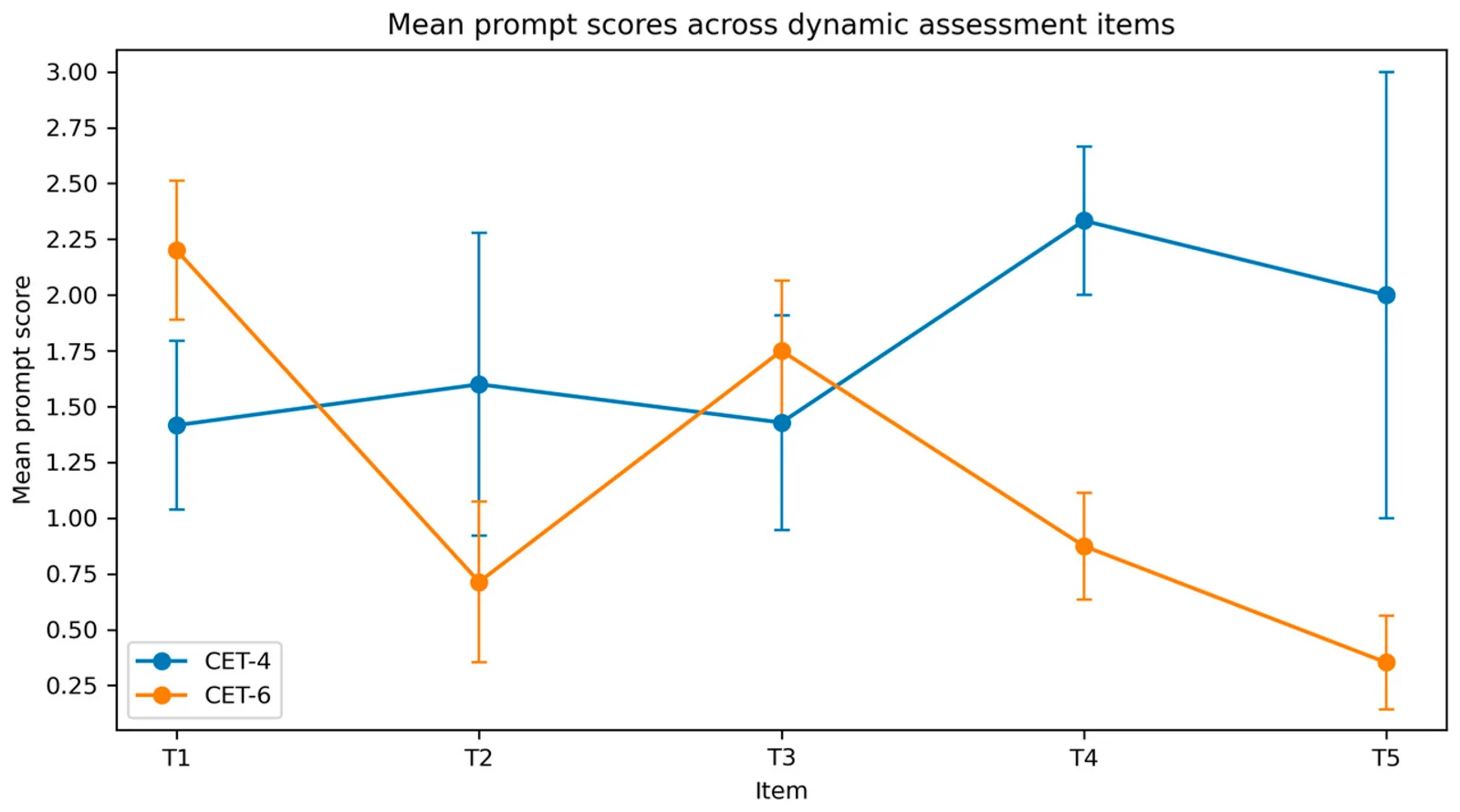
Mean prompt scores across dynamic assessment items.

The mean prompt scores across dynamic assessment items showed different developmental patterns for the two difficulty levels. In CET-4 reading, prompt scores showed an upward trend, suggesting that participants tended to solve later items with less explicit support. In CET-6 reading, prompt scores showed a downward trend, indicating that participants increasingly required more explicit prompts and that learning potential may have been less effectively activated under the higher-difficulty condition (Figure 4).

### 3.2. Correlations Among Behavioral Indicators

Pearson correlation analyses showed that actual scores were significantly negatively correlated with prompt scores in both CET-4 and CET-6 tasks (CET-4: r = −0.782, *p* < 0.001; CET-6: r = −0.611, *p* = 0.002). This indicates that learners with lower independent performance but potential for development benefited more from mediated prompts. Actual scores were significantly positively correlated with mediated scores in both tasks (CET-4: r = 0.886, *p* < 0.001; CET-6: r = 0.799, *p* < 0.001), suggesting that learners with stronger baseline ability retained an advantage in overall performance. LPS was highly correlated with mediated scores in CET-4 reading (r = 0.997, *p* < 0.001), but the corresponding relation was weak in CET-6 reading, indicating that mediation was more effectively translated into observable learning-potential gains under the moderate-difficulty condition (Table 2).

### 3.3. Effects of Reading Difficulty on Cortical Activation

**Figure 5:**
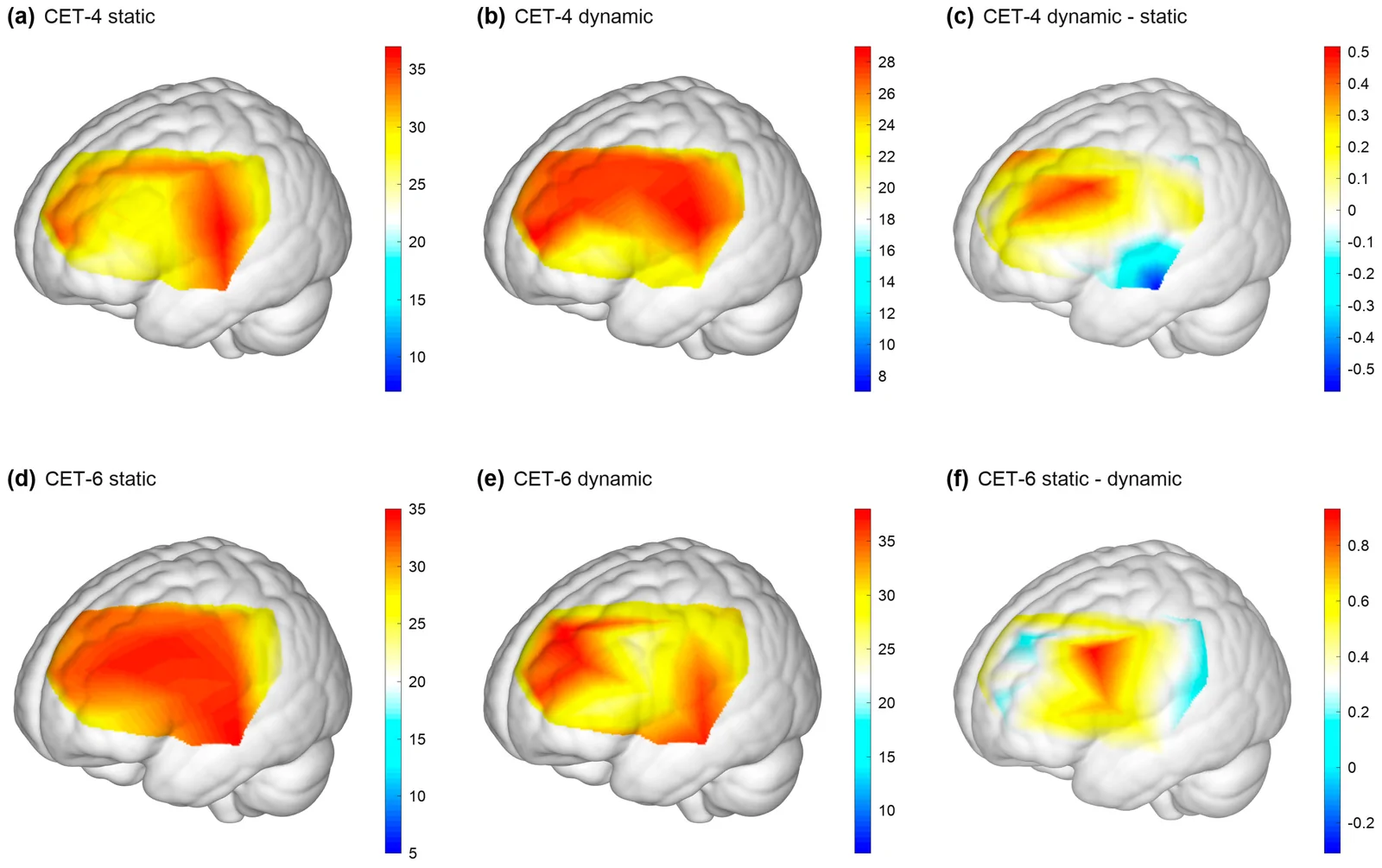
Activation-frequency maps and normalized activation-frequency difference maps for CET-4 and CET-6 static assessment, dynamic assessment, and static–dynamic comparisons. (**a**) CET-4 static; (**b**) CET-4 dynamic; (**c**) CET-4 normalized activation-frequency difference (dynamic minus static); (**d**) CET-6 static; (**e**) CET-6 dynamic; (**f**) CET-6 normalized activation-frequency difference (static minus dynamic).

The static-assessment summaries showed relatively high activation-frequency counts in monitored left frontal and temporal regions for both CET-4 and CET-6. In the CET-4 static assessment, AF1, T7, C3, AF3, F1, and C5 showed relatively high activation frequencies. In the CET-6 static assessment, AF1, F3, Fz, FC5, T7, C3, AF3, F1, FC3, C5, and FT7 showed relatively high activation frequencies. The CET-6 reading showed relatively high activation-frequency counts in more monitored regions than the CET-4 reading, particularly in areas corresponding to the DLPFC, MTG, MFG, and IFG, which are associated with executive control, semantic processing, and language comprehension (Table 3; Figure 5). This pattern suggests that the higher-difficulty CET-6 task may involve greater recruitment of cognitive-control and language-related resources, providing preliminary process-level information for the use of dynamic assessment in English reading comprehension.

### 3.4. Effects of Mediated Prompts on Cortical Activation

In the CET-4 condition, the normalized activation-frequency difference map (dynamic minus static) showed positive values in areas corresponding to the MFG and IFG, indicating higher condition-normalized activation-frequency values during dynamic than static assessment. The MFG is involved in working memory, attentional control, error monitoring, and decision making (Hamann & Carstengerdes, 2022), whereas the IFG is closely related to semantic processing and language control (Luo et al., 2025). This frequency-based pattern may therefore be associated with learners’ reanalysis of errors, extraction of textual cues, and strategic processing during mediated reattempts; however, these cognitive processes were not directly measured, and the pattern does not demonstrate stronger regional activation.

In the CET-6 condition, the normalized activation-frequency difference map (static minus dynamic) showed positive values in areas corresponding to the PreCG and IFG, indicating higher condition-normalized activation-frequency values during static than dynamic assessment. Combined with the behavioral pattern of decreasing prompt scores, this suggests that the higher-difficulty task may have exceeded the ZPD of some learners. Participants may have already invested substantial cognitive effort during the static phase; subsequently, task familiarity, fatigue, and limited prompt effectiveness are possible explanations for the lower dynamic-phase activation frequency, but these alternatives cannot be distinguished by the present analysis. This pattern suggests that behavioral responsiveness to mediation may vary across difficulty levels and may depend on the match between task difficulty and the learner’s current ability.

## 4. Discussion

### 4.1. Reading Difficulty, Cognitive Load, and English Reading Comprehension

The present findings show that top-ranked channels were identified in the monitored left prefrontal and temporal regions during both CET-4 and CET-6 reading, supporting the view that English reading comprehension requires executive control, working memory, and semantic processing. The DLPFC plays an important role in cognitive load and language processing (Butler et al., 2023), the MTG supports semantic cognition and sentence comprehension (Binder & Desai, 2011), and the IFG is involved in semantic selection, language control, and word processing (Luo et al., 2025). The CET-6 reading condition showed relatively high activation frequencies across a larger set of monitored regions than the CET-4 condition, suggesting that increased reading difficulty may require greater mobilization of cognitive resources. This neural pattern was consistent with the behavioral results: independent performance was lower in CET-6 than in CET-4, confirming that the higher-difficulty task imposed greater demands on learners.

### 4.2. Mediated Prompts, the ZPD, and the Activation of Learning Potential

The key purpose of DA is not merely to increase scores but to infer learning potential from learners’ responsiveness to mediation (Brown & French, 1979). In this study, the CET-4 dynamic condition showed higher condition-normalized activation-frequency values than the static condition in areas corresponding to the MFG and IFG, together with an upward trend in prompt scores. The co-occurrence of these patterns is consistent with the possibility that appropriately difficult tasks support effective use of mediated prompts. In other words, CET-4 reading was more likely to fall within the ZPD of most participants, enabling them to transform external support into internal strategies (Vygotsky, 1978).

In contrast, although average CET-6 scores improved after prompts, prompt scores decreased, and condition-normalized activation-frequency values in several monitored regions were lower during dynamic than static assessment. This suggests that improvement under the high-difficulty condition may have reflected local correction or task familiarity rather than stable activation of learning potential. The finding highlights the contextual and task-dependent nature of learning potential (Dixon et al., 2023). Mediation is most effective when the distance between task difficulty and the learner’s current level is developmentally appropriate (Tzuriel, 2000).

The present findings suggest that mediation should be interpreted together with task difficulty. A prompt does not have the same meaning across all difficulty levels. When the task is moderately challenging, as in the CET-4 condition for many participants, mediation may help learners use textual cues and revise their responses with relatively less external support. When the task is too difficult, as the CET-6 condition appeared to be for some participants, prompts may still improve some answers but may not be sufficient to produce stable behavioral responsiveness or sustained cortical engagement. This pattern is consistent with the DA assumption that learning potential is most observable when task demands are close to the learner’s ZPD.

### 4.3. Implications for Intelligence Assessment and Individualized Instruction

The theoretical contribution of this study is that it extends DA by considering behavioral measurement together with supplementary neurocognitive evidence. The Journal of Intelligence is concerned with human intelligence, cognitive development, assessment methods, and neuroimaging evidence. The present study treats English reading comprehension as a complex academic cognitive performance and uses fNIRS to examine cortical activity associated with task difficulty and mediated reattempts. The observed frontal and temporal activation-frequency patterns may be considered in relation to the parieto-frontal integration theory (P-FIT), which links intelligence to coordinated processing across distributed brain regions (Haier et al., 2023). Because the present montage covered only left frontal and temporal regions, this interpretation is preliminary and does not test the full P-FIT network. The findings do not imply that learning potential is directly measured or defined by brain activation. Rather, learning potential was operationalized through behavioral responsiveness to mediation, whereas fNIRS was used to provide supplementary process-level information.

Practically, the findings suggest that teachers should select reading materials according to students’ current levels and ZPDs and design prompts that move gradually from implicit to explicit support (Vygotsky, 1978). For tasks within the ZPD, prompts may help learners mobilize working memory, semantic processing, and strategy monitoring. For tasks far beyond the current level, teachers may need to reduce task difficulty, provide background knowledge, or decompose task steps before mediation can effectively activate learning potential.

### 4.4. Limitations and Future Directions

Several limitations should be noted. First, the valid sample size was small, and the gender distribution was uneven. Because the sample consisted of 18–30-year-old non-English-major university students with an English reading vocabulary of above 4000 words, the findings should be generalized primarily to learners with similar educational and proficiency backgrounds. Future research should examine whether the same mediation patterns appear in younger learners, English majors, lower-proficiency learners, and more diverse institutional contexts. Second, this study focused mainly on left frontal and temporal regions; future research could expand optode coverage to bilateral language networks and broader executive-control networks (Chan et al., 2022). Third, the present analysis used channel activation indices and activation frequencies to describe cortical activity. Future studies could apply general linear models, functional connectivity analysis, brain network metrics, and machine-learning approaches to improve the precision of neural interpretation (Akincioglu et al., 2025). Fourth, although CET-4 and CET-6 materials provide standardized and ecologically valid difficulty levels, future work should further match text length, vocabulary difficulty, syntactic complexity, and question type to separate language difficulty from task-format effects. Fifth, dynamic-phase observations were available only for items answered incorrectly in the static phase. The two phases were therefore based on different sets of observations, and the activation-frequency comparison maps should be interpreted descriptively.

## 5. Conclusions

This study used fNIRS to examine cortical activation during English reading comprehension tasks of different difficulty levels under static and dynamic assessment conditions. Behavioral scores were analyzed together with mediated prompt responses to evaluate learning potential. The results showed that both CET-4 and CET-6 reading tasks activated the DLPFC and MTG, which are associated with executive control and semantic processing. Compared with CET-4 reading, CET-6 reading elicited broader activation in language- and cognitive-control-related regions, indicating that higher-difficulty reading required greater cognitive resources. Mediated prompts were associated with both activation-frequency patterns and behavioral responsiveness. In the CET-4 reading test, dynamic assessment showed higher normalized activation-frequency values than static assessment in areas corresponding to the MFG and IFG, and prompt scores showed an upward trend. In the CET-6 reading test, static assessment showed higher normalized activation-frequency values than dynamic assessment in areas corresponding to the PreCG and IFG, and prompt scores showed a downward trend. These findings suggest that mediated prompts are more effective in activating learning potential under appropriately challenging conditions, whereas excessive difficulty may constrain mediation.

Overall, the study provides preliminary neurocognitive evidence for the dynamic assessment of foreign-language reading comprehension. It provides preliminary evidence for the feasibility of using fNIRS as a supplementary process-level indicator beyond behavioral scores to describe cortical activity associated with task difficulty and mediated reattempts, rather than to directly define learning potential. The findings contribute to research on learning potential, academic cognition, and individualized instructional intervention. Practically, DA-style prompts may effectively help teachers identify the support learners need and adjust task difficulty before mediation.

## Figures and Tables

**Table 1:** Mediation level scale and prompt scoring rules.

Level	Mediated Prompt	Score If Correct After Prompt	Score If Incorrect After Prompt
1	Inform the learner that the answer is incorrect	3	0
2	Provide information related to the correct answer	2	0
3	Provide more explicit information related to the correct answer	1	0

**Table 2:** Correlations among behavioral indicators in CET-4 and CET-6 reading.

Measure	Statistic	CET-4 Actual	CET-4 Prompt	CET-4 Mediated	LPS4	CET-6 Actual	CET-6 Prompt	CET-6 Mediated	LPS6
Prompt score	Pearson	−0.782 **	1			−0.611 **	1		
	Sig.	0.000				0.002			
Mediated score	Pearson	0.886 **	−0.403	1		0.799 **	−0.012	1	
	Sig.	0.000	0.057			0.000	0.958		
LPS	Pearson	0.910 **	−0.456 *	0.997 **	1	0.103	−0.135	0.027	1
	Sig.	0.000	0.029	0.000		0.641	0.540	0.903	

Note. ** *p* < 0.01, two-tailed; * *p* < 0.05, two-tailed.

**Table 3:** Activation-frequency counts of cortical regions in static and dynamic assessments.

Region/Electrode	CET-4 Static	CET-4 Dynamic	CET-6 Static	CET-6 Dynamic
AF5	8	13	15	18
AF1	27	22	27	26
F3	19	26	33	35
Fz	14	22	26	14
FC5	18	17	30	13
T7	31	12	35	36
C3	29	26	28	18
CP5	13	17	5	16
F5	7	7	22	9
AF3	30	29	26	34
AFz	14	17	20	6
F1	24	25	27	38
FC3	16	25	33	6
C5	37	29	31	32
FT7	18	13	27	30
CP3	13	9	13	21

## Data Availability

The data presented in this study are available from the corresponding author upon reasonable request. Public sharing of the data is restricted because the dataset contains sensitive physiological information from student participants.

## Abbreviations

The following abbreviations are used in this manuscript:

fNIRS	functional near-infrared spectroscopy
CET-4	College English Test Band 4
CET-6	College English Test Band 6
DA	dynamic assessment
ZPD	zone of proximal development
HbO	oxygenated hemoglobin
HbR	deoxygenated hemoglobin
DLPFC	dorsolateral prefrontal cortex
MFG	middle frontal gyrus
IFG	inferior frontal gyrus
PreCG	precentral gyrus
MTG	middle temporal gyrus

## Appendix A

**Table A1:** MNI Coordinates of Optodes and Corresponding Brain Regions.

Optode	Region	X (mm)	Y (mm)	Z (mm)	Brodmann Area	Corresponding Cortical Region
S1	AF5	−45.336	73.867	−0.879	BA46	DLPFC
S2	AF1	−19.609	82.143	27.460	BA46	DLPFC
S3	F3	−51.830	55.753	37.164	BA9	MFG
S4	Fz	−0.235	62.434	65.513	BA9/46	MFG/DLPFC
S5	FC5	−77.435	19.001	18.879	BA44/45	IFG
S6	T7	−84.830	−16.239	−16.314	BA21	MTG
S7	C3	−70.634	−11.463	63.077	BA4	PreCG
S8	CP5	−82.542	−47.309	27.800	BA40	Supramarginal gyrus
D1	F5	−64.987	49.590	10.244	BA9	MFG
D2	AF3	−35.609	78.819	16.092	BA46	DLPFC
D3	AFz	−0.040	83.390	31.683	BA46	DLPFC
D4	F1	−29.480	60.438	57.351	BA9	MFG
D5	FC3	−63.100	23.879	52.682	BA44/45	IFG
D6	C5	−84.098	−13.846	24.756	BA4	PreCG
D7	FT7	−79.225	13.769	−18.190	BA21	MTG
D8	CP3	−69.286	−48.011	65.668	BA40	Supramarginal gyrus

**Table A2:** MNI Coordinates of Channels.

Channel	X (mm)	Y (mm)	Z (mm)	Depth (mm)
1 (S1-D1)	−56.290	62.945	5.070	33.2
2 (S1-D2)	−40.271	76.807	7.216	20.2
3 (S2-D2)	−27.888	81.255	21.645	19.9
4 (S2-D3)	−9.708	83.305	30.011	20.1
5 (S2-D4)	−25.784	72.446	43.739	38.2
6 (S3-D1)	−58.802	54.143	23.513	30.6
7 (S3-D2)	−44.937	68.817	26.355	35.2
8 (S3-D4)	−41.387	58.420	48.855	30.5
9 (S3-D5)	−58.817	39.436	45.984	37.2
10 (S4-D3)	0.193	75.250	49.430	39.8
11 (S4-D4)	−15.837	63.087	62.131	30.4
12 (S5-D1)	−72.412	34.597	14.859	34.1
13 (S5-D5)	−71.835	22.115	37.640	37.0
14 (S5-D6)	−81.342	2.605	21.617	34.0
15 (S5-D7)	−78.803	16.889	0.924	37.5
16 (S6-D6)	−85.287	−14.179	3.539	41.1
17 (S6-D7)	−82.152	−0.704	−17.684	30.6
18 (S7-D5)	−67.497	6.477	58.072	37.6
19 (S7-D6)	−80.047	−11.793	44.030	40.7
20 (S7-D8)	−72.341	−29.617	65.522	36.7
21 (S8-D6)	−85.028	−29.926	25.727	33.6
22 (S8-D6)	−78.594	−47.958	46.962	40.1

## References

[B1-jintelligence-14-00180] Ajideh P., Nourdad N. (2012). The effect of dynamic assessment on EFL reading comprehension in different proficiency levels. Language Testing in Asia.

[B2-jintelligence-14-00180] Akincioglu U., Aydemir O., Cil A., Baydere M. (2025). An ICEEMDAN and SAX-based method for determining English reading comprehension status using functional near-infrared spectroscopy signals. PLoS ONE.

[B3-jintelligence-14-00180] Aziz M., Rawian R. (2022). Modeling higher order thinking skills and metacognitive awareness in English reading comprehension among university learners. Frontiers in Education.

[B4-jintelligence-14-00180] Babayiğit S., Hitch G. J., Kandru-Pothineni S., Clarke A., Warmington M. (2022). Vocabulary limitations undermine bilingual children’s reading comprehension despite bilingual cognitive strengths. Reading and Writing.

[B5-jintelligence-14-00180] Behbahani H. K., Karimpour S. (2025). Exploring the role of mobile-mediated dynamic assessment in L2 learners’ reading comprehension and reading fluency. Education and Information Technologies.

[B6-jintelligence-14-00180] Binder J. R., Desai R. H. (2011). The neurobiology of semantic memory. Trends in Cognitive Sciences.

[B7-jintelligence-14-00180] Brown A. L., French L. A. (1979). The zone of potential development: Implications for intelligence testing in the year 2000. Intelligence.

[B8-jintelligence-14-00180] Butler L. K., Pecukonis M., Rogers D. J., Boas D. A., Tager-Flusberg H., Yücel M. A. (2023). The role of the dorsolateral prefrontal cortex in the production and comprehension of phonologically and semantically related words. Brain Sciences.

[B9-jintelligence-14-00180] Chan M. M. Y., Chan M. C., Yeung M. K., Wang S. M., Liu D., Han Y. M. Y. (2022). Aberrant prefrontal functional connectivity during verbal fluency test is associated with reading comprehension deficits in autism spectrum disorder: An fNIRS study. Frontiers in Psychology.

[B10-jintelligence-14-00180] Čeko M., Hirshfield L., Doherty E., Southwell R., D’Mello S. K. (2024). Cortical cognitive processing during reading captured using functional-near infrared spectroscopy. Scientific Reports.

[B11-jintelligence-14-00180] Da Silva Soares R., Ambriola Oku A. Y., Barreto C. S. F., Ricardo Sato J. (2022). Applying functional near-infrared spectroscopy and eye-tracking in a naturalistic educational environment to investigate physiological aspects that underlie the cognitive effort of children during mental rotation tests. Frontiers in Human Neuroscience.

[B12-jintelligence-14-00180] Dixon C., Oxley E., Gellert A. S., Nash H. (2023). Dynamic assessment as a predictor of reading development: A systematic review. Reading and Writing.

[B13-jintelligence-14-00180] Haier R. J., Colom R., Hunt E. (2023). The science of human intelligence.

[B14-jintelligence-14-00180] Hamann A., Carstengerdes N. (2022). Investigating mental workload-induced changes in cortical oxygenation and frontal theta activity during simulated flights. Scientific Reports.

[B15-jintelligence-14-00180] He Y., Hu Y. (2022). Functional connectivity signatures underlying simultaneous language translation in interpreters and non-interpreters of mandarin and English: An fNIRS study. Brain Sciences.

[B16-jintelligence-14-00180] Jia L., Cai J., Wang J. (2023). Promoting learning potential among students of L2 Chinese through dynamic assessment. Language Assessment Quarterly.

[B17-jintelligence-14-00180] Jiang R., Xiao Z., Jiang Y., Jiang X. (2025). The neural mechanisms of private speech in second language learners’ oral production: An fNIRS study. Brain Sciences.

[B18-jintelligence-14-00180] Jin C., Liu Y. (2026). Diagnosing and promoting learners’ L2 inferential reading development through hybrid computerised dynamic assessment in the Chinese EFL classroom. Computer Assisted Language Learning.

[B19-jintelligence-14-00180] Jöbsis F. F. (1977). Noninvasive, infrared monitoring of cerebral and myocardial oxygen sufficiency and circulatory parameters. Science.

[B20-jintelligence-14-00180] Kozulin A., Garb E. (2003). Dynamic assessment of EFL text comprehension. School Psychology International.

[B21-jintelligence-14-00180] Li J., Li Y., Wang H., Chen J., Li R., Zhang H. (2025). Effect of teacher–student interaction on language learning under anxiety: An fNIRS-based hyperscanning study. Language Learning.

[B22-jintelligence-14-00180] Luo H., Yu T., Li Q., Sheng L. (2025). Functional specialization for language processing in inferior frontal regions during early childhood: Evidence from functional near-infrared spectroscopy individual functional channels of interest approach. Neurophotonics.

[B23-jintelligence-14-00180] Marks R. A., Sun X., McAlister López E., Nickerson N., Hernandez I., Caruso V. C., Satterfield T., Kovelman I. (2022). Cross-linguistic differences in the associations between morphological awareness and reading in Spanish and English in young simultaneous bilinguals. International Journal of Bilingual Education and Bilingualism.

[B24-jintelligence-14-00180] Piyumi Udeshinee W. A., Knutsson O., Barbutiu S. M., Jayathilake C. (2024). Re-designing a regulatory scale for dynamic assessment in the synchronous text chat environment in collaboration with teachers. Computer Assisted Language Learning.

[B25-jintelligence-14-00180] Poehner M. E., Lantolf J. P. (2013). Bringing the ZPD into the equation: Capturing L2 development during computerized dynamic assessment (C-DA). Language Teaching Research.

[B26-jintelligence-14-00180] Poehner M. E., Wang Z. (2021). Dynamic assessment and second language development. Language Teaching.

[B27-jintelligence-14-00180] Poehner M. E., Zhang J., Lu X. (2015). Computerized dynamic assessment (C-DA): Diagnosing L2 development according to learner responsiveness to mediation. Language Testing.

[B28-jintelligence-14-00180] Rovetti J., Goy H., Nurgitz R., Russo F. A. (2021). Comparing verbal working memory load in auditory and visual modalities using functional near-infrared spectroscopy. Behavioural Brain Research.

[B29-jintelligence-14-00180] Siengyen C., Wasanasomsithi P. (2024). Development of a computerized dynamic reading assessment program to measure English reading comprehension of Thai EFL undergraduate students. rEFLections.

[B30-jintelligence-14-00180] Sun Z., Xu P., Wang J. (2023). Dynamic assessment of the learning potential of Chinese as a second language. Language Assessment Quarterly.

[B31-jintelligence-14-00180] Teo A. (2014). Beyond traditional testing: Exploring the use of computerized dynamic assessment to improve EFL learners’ reading. Arab World English Journal.

[B32-jintelligence-14-00180] Tzuriel D. (2000). Dynamic assessment of young children: Educational and intervention perspectives. Educational Psychology Review.

[B33-jintelligence-14-00180] Vygotsky L. S. (1978). Mind in society: The development of higher psychological processes.

[B34-jintelligence-14-00180] Yang Y., Qian D. D. (2017). Assessing English reading comprehension by Chinese EFL learners in computerized dynamic assessment. Language Testing in Asia.

[B35-jintelligence-14-00180] Yang Y., Qian D. D. (2023). Enhancing EFL learners’ reading proficiency through dynamic assessment. Language Assessment Quarterly.

[B36-jintelligence-14-00180] Zadkhast M., Rezvani E., Lotfi A. R. (2023). Effects of concurrent and cumulative group dynamic assessments on EFL learners’ development of reading comprehension micro-skills. Language Testing in Asia.

[B37-jintelligence-14-00180] Zhang K., Chen J., Yang Z. (2024). Use of human–computer interactive games for the dynamic assessment of language skills of children with autism spectrum disorder. British Journal of Educational Technology.

[B38-jintelligence-14-00180] Zhao H., Aryadoust V. (2025). A meta-analysis of the reliability of second language reading comprehension assessment tools. Studies in Second Language Acquisition.

